# Body mass index across adulthood and the development of airflow obstruction and emphysema

**DOI:** 10.1177/14799731221139294

**Published:** 2022-11-09

**Authors:** Ruth E Trethewey, Nicole L Spartano, Ramachandran S Vasan, Martin G Larson, George T O’Connor, Dale W Esliger, Emily S Petherick, Michael C Steiner

**Affiliations:** 1 School of Sport, Exercise and Health Sciences, 5156 Loughborough University, Loughborough, UK; 2 National Centre for Sport and Exercise Medicine, 5156 Loughborough University, Loughborough, UK; 3 Lung and Blood Institute’s Framingham Heart Study, 1846 Boston University and National Heart, Framingham, MA, USA; 4 Centre for Exercise and Rehabilitation Services, Leicester, UK; 5 NIHR Leicester Biomedical Research Centre––Respiratory, 4488 University of Leicester, Leicester, UK

**Keywords:** Airway obstruction, BMI, body composition, COPD, lean mass, lung pathology

## Abstract

**Background:**

Low body mass index (BMI) is associated with COPD, but temporal relationships between airflow obstruction (AO) development and emphysematous change are unclear. We investigated longitudinal changes in BMI, AO, and lung density throughout adulthood using data from the Framingham Offspring Cohort (FOC).

**Methods:**

BMI trajectories were modelled throughout adulthood in 4587 FOC participants from Exam 2 (mean age = 44), through Exam 9 (mean age = 71), in AO participants and non-AO participants (AO *n* = 1036), determined by spirometry, using fractional polynomial growth curves. This process was repeated for low lung density (LLD) and non LLD participants (LLD *n* = 225) determined by Computed Tomography. Spirometry decline was compared separately between tertiles of BMI in those aged <40 years and associations between fat and lean mass (measured using Dual Energy X-ray Absorptiometry, DEXA) and development of AO and LLD were also assessed. Additional analyses were performed with adjustment for smoking volume.

**Results:**

The BMI trajectory from 30 years of age was visually lower in the AO group than both non-AO smokers (non-<AO-S) and non-AO non-smokers (non-AO-N). Similarly, BMI trajectories were visually lower in participants with LLD throughout adulthood compared to normal lung density smokers and non-smokers. Differences remained after adjustment for smoking volume. The lowest BMI tertile in ages <40 years was associated with the steepest subsequent decline in FEV_1_/FVC ratio in both sexes.

**Conclusion:**

Mean BMI is lower throughout adulthood in AO and LLD participants. Lower BMI is associated with a steeper decline in the ratio of FEV_1_/FVC. These findings suggest body mass may precede and potentially have a role in the development of COPD lung pathophysiology.

## Introduction

Weight loss and low body mass index (BMI) have a recognised association with Chronic Obstructive Pulmonary Disease (COPD).^1–3^ The presence of a low BMI or weight loss in individuals with COPD compromises health status and exercise capacity, and predicts morbidity and mortality independently of lung function impairment.^4–7^ A decline in body mass in COPD has conventionally been considered a consequence of the disease, attributable to factors such as systemic inflammation, reduced calorie intake and increased energy expenditure.^8,9^ Cross-sectional and short-term observational studies suggest low body mass in earlier life is associated with development of airflow obstruction suggesting a potential role for body habitus and smaller tissue reserves in COPD pathophysiology in conjunction with smoking or other noxious stimuli.^10–12^ Other studies have highlighted the impact of obesity and weight gain on lung function decline but not the development of airflow obstruction.^13,14^ Most studies have been limited in follow-up duration to examine the association of BMI over the life-course with the subsequent development of airflow obstruction and few have corroborated such associations with changes in lung structure and measures of lean mass.

We addressed this using data from the Framingham Offspring Cohort, which recorded indices of lung function/structure and body habitus (BMI and lean tissue mass) over four decades, and ages spanning across adulthood. These data provide a unique opportunity to examine temporal relationships between BMI and the development of lung functional and structural impairment in support of the hypothesis that lower BMI and therefore tissue reserves are associated with COPD pathogenesis.

Four separate analyses were performed, collectively allowing the triangulation of observations in support of the hypothesis that body mass is implicated in the aetiology of decline in lung function and structure.^15^

(i) To determine whether BMI is lower in earlier adulthood in those who developed airflow obstruction, BMI trajectories were modelled across adulthood in participants who either exhibited airflow obstruction or did not according to spirometry measures performed throughout study follow up.
(ii) To determine whether BMI is lower in earlier adulthood in those who exhibited low lung density, BMI trajectories were modelled across adulthood in participants who exhibited low lung density or did not at computed tomography (CT) scan, performed at a single time point in the study follow up.
(iii) To investigate whether there is a steeper age-related decline in spirometry measurements, particularly the FEV_1_/FVC ratio, with lower BMIs the change in spirometry indices with age in different BMI tertiles was analysed.
(iv) Associations between fat or lean mass (measured using Dual Energy X-ray Absorptiometry, DEXA) and development of airflow obstruction or low lung density were also assessed.

## Methods

### Cohort design

The Framingham Offspring Cohort comprises offspring and spouses from the original Framingham Heart Study, a prospective longitudinal cohort examining cardiovascular disease in adults living in the town of Framingham, Massachusetts. The Offspring cohort began with Exam 1 in 1971 when 5124 participants were recruited with ages spanning between 5 and 70 years. Participant follow-up has occurred at approximately four yearly intervals to the present, as previously described.^16^ There was an initial high dropout between Exam 1 and Exam 2, but no further recruitment took place. Participants did not necessarily attend all exams and may have attended Exam 3 without attending Exam 2 for example. Our analyses included all participants who attended between Exam 2 to Exam 9, which is the latest available. Exam 1 was not included due to the high dropout between Exam 1 and Exam 2 which may otherwise skew results. Participants underwent a detailed medical assessment and clinical examination at every exam and in addition underwent additional investigations such as DEXA and chest CT.

Inclusion Criteria for the analyses are provided in the supplement.

Full details of measurement methods are provided in the supplement.

### Operational definitions

Airflow obstruction was defined as a ratio of FEV_1_/FVC below the lower limit of normal using the Global Lung Initiative (GLI) reference ranges, and an FEV_1_ below 80% predicted.^17^ This definition was chosen in order to reduce the overdiagnosis of AO with age, which is particularly relevant to a population level study.^17^ *Low lung density* was defined as the presence of greater than 5% of the total lung with a density of below −950 Hounsfield units on CT. For the initial analysis smoking status was divided into “ever-smokers” and “never-smokers.” “Ever-smokers” were defined as those who reported either being an ex-smoker or current smoker at any of the exams. Pack years were calculated as number of cigarettes/20 × number of years.

### Statistical analysis

**Figure 1.**
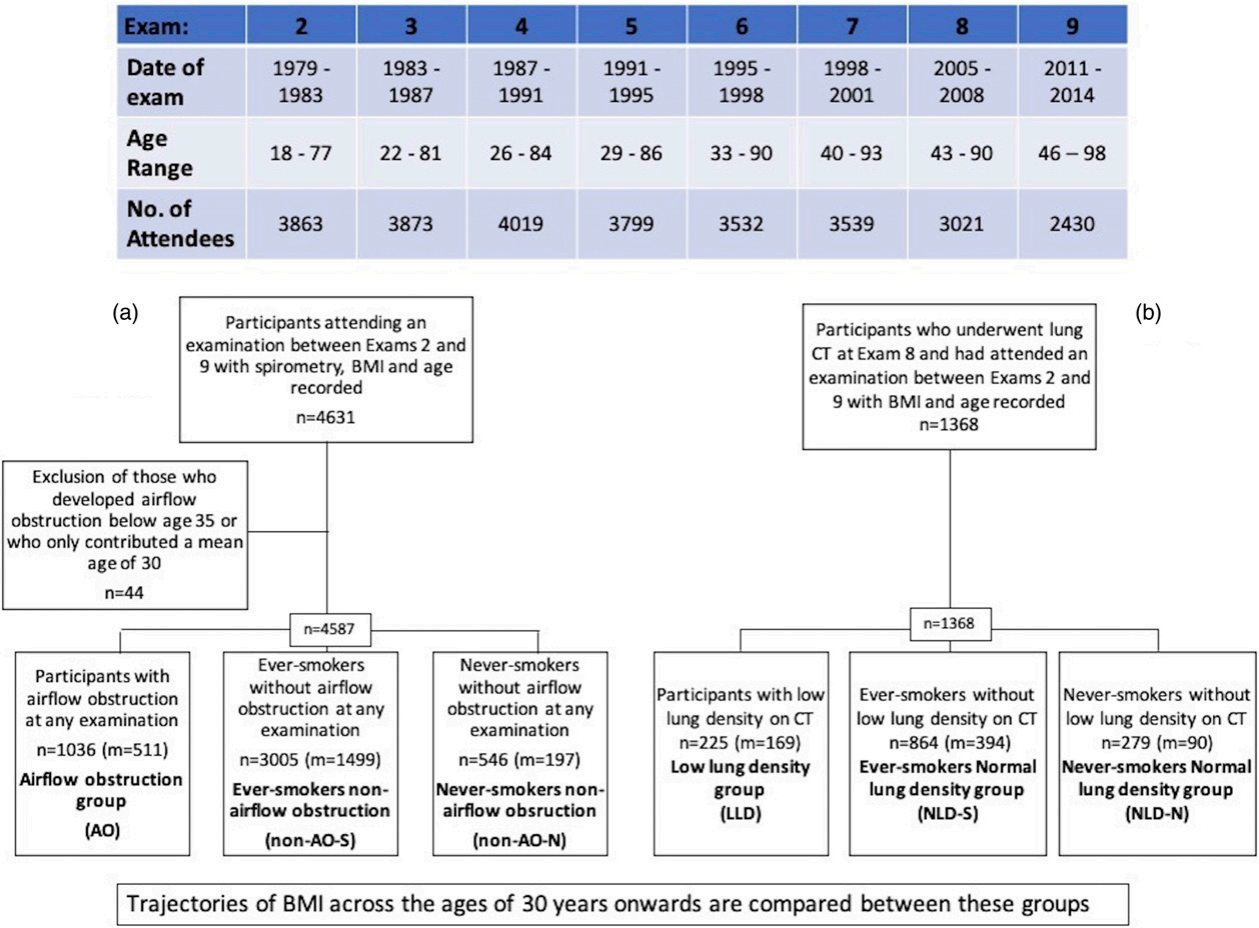
Flow chart of participant inclusion and group allocation for the four different analyses used to examine the relationship between BMI and spirometry indices (a) and lung density (b). n: number of participants; m: men; BMI: body mass index; CT: computed tomogram.

(i) The modelling of BMI trajectories across adulthood in participants who either exhibited incident airflow obstruction or not according to spirometry measures performed throughout study follow up:

To examine differences in BMI in earlier adulthood between participants with or without incident airflow obstruction (AO), BMI trajectories using fractional polynomial models across adulthood were plotted. These models used data from examination cycle 2 (1979–1983) to examination cycle 9 (2011–2014) in groups determined by whether participants did or did not exhibit airflow obstruction during the study period: “airflow obstruction” (AO), “non-airflow obstruction never smokers” (non-AO-N) and “non-airflow obstruction ever-smokers” (non-AO-S). Participants contributed to the data at the age they entered the study. This was done so that the trajectory of BMI over time could be most accurately captured in each group. Smokers were modelled separately in non-AO groups in view of known effects of smoking on lung function, but insufficient numbers of non-smokers meant that division of AO groups was not possible. Participant inclusion and group allocation is shown in Figure 1.

(ii) The modelling of BMI trajectories in participants who either had low lung density at CT scan or not, performed at a single time point in the study follow up:

Similarly to AO groups BMI trajectories using fractional polynomial models were plotted for groups determined by the presence or absence of low lung density (LLD) at quantitative computed tomography (CT) performed later in the cohort (between 2002 and 2005): “LLD group,” “non-LLD ever-smokers (NLD-S) and “non-LLD never-smokers” (NLD-N). Insufficient numbers of non-smokers meant that division of LLD groups was not possible. Participant inclusion and group allocation is shown in Figure 1.

Further details on analysis for objectives (i) and (ii) are located in the supplement. Conclusions from these analyses were drawn from visual inspection of the models.

(iii) Analysing the change in spirometry indices with age between BMI tertiles:

Whether lower BMI was associated with faster subsequent lung function decline in participants with recordings of BMI and spirometry before the age of 40 (i.e., prior to the onset of the majority of cases of COPD) was assessed. Changes in FEV_1_ (forced expiratory volume in 1s), FVC (forced vital capacity) and FEV_1_/FVC ratio with age were compared in a stratified, descriptive analysis of different BMI tertiles (low, middle and high) using multiple linear regression. BMI tertiles were chosen due to the unequal distribution of participants between standard BMI categories. To standardise values, the percent of age 25 predicted (“%age25”) FEV_1_ and FVC values were used in the analysis of change in spirometry measures. These were calculated as the measured value divided by the predicted value for the individual at age 25 years (i.e., predicted peak lung function) using the Global Lung Initiative reference value, presented as a percentage. The ratio of FEV_1_/FVC was calculated using the absolute values. In these calculations ethnicity was assumed to be “white” due to its predominance within the cohort.^18^ Height was adjusted for within the analysis and men and women modelled separately in view of differences in spirometry decline between sexes. Further details are included in the supplementary material.

(iv) Assessing associations between fat or lean mass and the development of airflow obstruction or low lung density:

Triangulation of the earlier findings was performed by investigating the relationship between lean mass index and fat mass index measured by DEXA with the outcome of either the presence of airflow obstruction at Exam 8 or low lung density on CT was assessed using logistic regression as described in the supplemental material.

A sensitivity analysis was undertaken for a subsample of the cohort with complete smoking pack year data, repeating BMI trajectory and spirometry decline models adjusting for effects of smoking volume discrepancies on BMI. Details of this analysis are within the supplement.

It is not possible to present true baseline characteristics at a specific age due to the nature of the data recording and the wide age range at each examination. Characteristics for AO and LLD groups at Exam 2 for participants who attended this examination were presented as mean and standard deviation for continuous variables and counts and percentages for categorical variables (Tables 1 and 2). The frequency of airflow obstruction for the different age categories for each exam and for the whole cohort is presented in Figure S1 in the supplementary material. Differences in baseline characteristics for groups determined by the presence of AO and LLD were analysed using independent t-tests or one-way analysis of variance (ANOVA) for continuous data and Pearson Chi squared for categorical data. Statistical significance was defined as *p* value <0.05. Analyses were performed using Stata version 14 (StataCorp.2015) and MLWin version 2.36 (Centre for Multilevel Modelling, University of Bristol, 2016), using the runmlwin command in Stata.^19^

**Table 1.** Participant characteristics recorded at Exam 2 according to later development of airflow obstruction.

	Non-airflow obstruction		Airflow obstruction	
	Men	Women	Men	Women
*N*	1504	1640	332	330
Age (years), mean SD	45.1 (10.3)*	44.3 (9.9)*	47.5 (9.7)	46.3 (9.4)
BMI (kg/m^2^), mean SD	26.7 (3.7)*	24.7 (5.0)*	26.2 (3.7)	23.6 (3.7)
Height (m), mean SD	1.7 (0.1)	1.6 (0.1)	1.7 (0.1)	1.6 (0.1)
Pack years^†^	18.3 (22.0)*	10.1 (15.0)	31.7 (27.9)	22.5 (20.2)
Smoking – number of smokers (%)	1330 (88.4)*	1332 (81.2)*	322 (97.0)	316 (95.8)
BMI categories (kg/m^2^) *n* (%)				
<18.5 (underweight)	7 (0.5)	37 (2.3)	2 (0.6)	12 (3.6)
18.5–25 (normal)	507 (33.7)	1020 (62.2)	126 (38.0)	221 (67.0)
25–30 (overweight)	745 (49.5)	367 (22.4)	160 (48.2)	76 (23.0)
30–35 (obese)	208 (13.8)	123 (7.5)	40 (12.1)	19 (5.8)
>35	37 (2.5)	93 (5.7)	4 (1.2)	2 (0.6)

Participants are divided into groups determined by the development of airflow obstruction at later exam cycles (i.e. any timepoint after initial enrolment at exam 2).

Data shown for subjects attending exam 2 only (i.e., 3806 out of 4587).

SD: standard deviation, n: number of participants, BMI: body mass index, m: metres, kg: kilograms, n: number, %: percentage. *Denotes statistical difference between values for airflow obstruction and non-airflow obstruction groups.

^†^Pack year data available for 4144 participants in this analysis.

**Table 2.** Participant characteristics recorded at Exam 2 according to later development of Low Lung Density on CT.

	Normal lung density		Low lung density	
	Men	Women	Men	Women
*N*	431	592	146	54
		Mean (SD)		
Age at exam 2 (years)	41.8 (9.2)	41.5 (8.9)*	42.1 (9.3)	46 (8.6)
BMI at exam 2 (kg/m^2^)	26.8 (3.6)*	24.2 (4.4)	25.4 (3.0)	23.3 (5.5)
Height at exam 2 (m)	1.7 (0.1)	1.6 (0.1)	1.7 (0.1)	1.6 (0.1)
Mean lung density (at scan date)	−855.5 (42.1)	−854.6 (45.0)	−879.1 (32.6)	−891.3 (27.2)
Pack years at exam 2^†^	13.2 (0.9)*	7.7 (0.5)*	16.9 (1.7)	18.8 (2.3)
Current smokers at exam 8 - n (%)	30 (6.9)*	38 (6.4)	5 (3.3)	3 (6.4)
Spirometric airflow obstruction––*n* (%)	104 (21.5)*	132 (20.1)*	109 (64.5)	32 (57.1)

Participants are divided into groups determined by the development of low lung density at later exam cycles (exam cycles after 2008).

Data shown for subjects attending exam 2 only (i.e., 1223 out of 1368).

SD: standard deviation, n: number of participants, BMI: body mass index, m: metres, kg: kilograms, %: percentage. *Denotes statistical difference between values for low lung density and normal lung density groups.

^†^Pack year data available for 1210 participants in this analysis.

### Ethical approval

Ethical approval for the study was provided by Loughborough University and permission to access the data was provided by the National Heart, Lung and Blood Institute (NHLBI).

## Results

Of the 5124 subjects that attended exam 1, 3863 subjects returned for exam 2, which was the largest drop in numbers within the cohort period. Some of these missing subjects returned for later exams so subject numbers fluctuated and were 4019 at exam 4 for example. While not every subject attended every examination (including Exam 2) attrition rates were low as seen in Figure 1(a).

### Objective (i): BMI across adulthood in AO and non-AO groups

Four thousand five hundred eighty-seven (83% of whom were present at Exam 2) subjects had relevant data and met inclusion criteria for this analysis. Participant characteristics at Exam 2, presented in Table 1, include a mean age of 45.2 years. AO groups were older than non-AO groups. BMI was higher in men than women and higher in non-AO groups than AO groups. Smoking was more prevalent in AO groups than non-AO groups. The spread of participants across BMI categories is seen in Table 1. The distribution of the frequency of airflow obstruction occurrences for each exam cycle and across the entire cohort is shown in Figure S1, with the peak incidence of AO for the cohort between 60 and 70 years.

**Figure 2.**
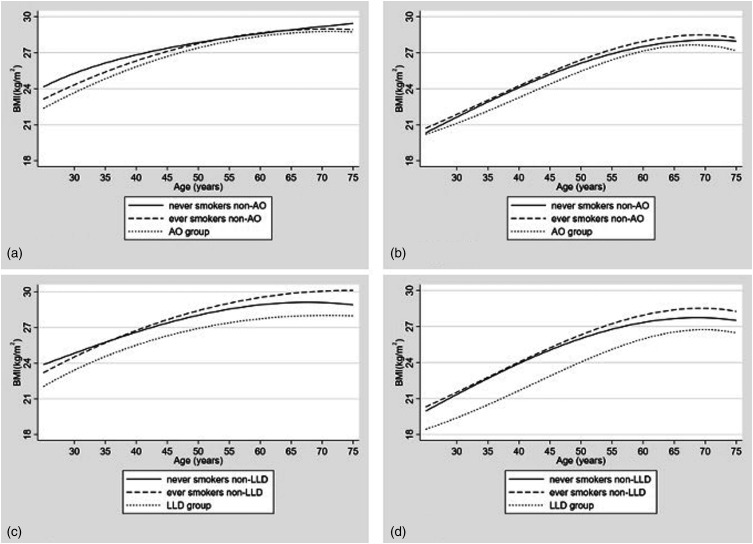
Fractional polynomial models for BMI across adulthood for those who did and did not develop airflow obstruction in (a) men and (b) women and for those with and without low lung density at lung CT in (c) men and (d) women. BMI: body mass index; CT: computed tomogram; kg: kilograms; m: metre.

Fractional polynomial powers for age for BMI trajectory models are provided in the supplemental material. In all groups BMI increased with age, levelling out towards the 7th decade. BMI was visually lower in AO groups than non-AO groups across all ages (Figure 2a and b). On visual inspection of the graphs for men, BMI was higher throughout most of adulthood in the non-AO-N than non-AO-S group but converged between ages 50 and 65 years (Figure 2a). In women, BMI appeared higher in the non-AO-S than the non-AO-N group (Figure 2b).

Knot points for all linear spline models were selected at ages 30, 45, 60 and 75 years based on visual inspection of fractional polynomial models. The values for predicted BMI from linear spline models were visually lower in AO groups than non-AO groups (Table S1).

### Objective (ii): BMI across adulthood for LLD and non-LLD

One thousand three hundred sixty-eight subjects (89% of whom were present at Exam 2) had relevant data and met inclusion criteria for this analysis. Characteristics at Exam 2 for participants later identified as LLD and non-LLD groups are shown in Table 2. Age was similar between groups in men, but the LLD group was older than non-LLD groups in women. A large proportion with LLD developed airflow obstruction (men: 64.5%, women: 57.1%). BMI was graphically lower in LLD groups than non-LLD groups across adulthood (Figure 2c and d). Knot points for all linear spline models were selected at ages 30, 45, 60 and 75 years. The numerical values for predicted BMI from linear spline models were visually marginally larger between groups determined by lung density than by airflow obstruction (Table 3).

**Table 3.** Values for predicted BMI derived from linear spline models for participants that developed and did not develop airflow obstruction and low lung density using spline models at age 35 and age 45.

	Predicted BMI age 35		Predicted BMI age 45	
	(BMI kg/m^2^)			
	Men	Women	Men	Women
Airflow obstruction (AO)	24.7	21.8	26.7	24.6
Ever-smokers non-airflow obstruction (non-AO-S)	25.3	23.0	27.1	25.5
Never-smokers non-airflow obstruction (non-AO-N)	25.9	22.9	27.3	25.5
Low lung density (LLD)	24.5	21.5	26.4	23.4
Ever-smokers normal lung density (NLD-S)	26.0	22.8	27.6	25.4
Never-smokers normal lung density (NLD-N)	25.7	22.9	27.6	25.1

The numeric values refer to points along splines corresponding to ages 35 and 45 for men and women. These points were chosen as representative of different points in adulthood. Values are provided for participants who do or do not develop both airflow obstruction and low lung density) subsequent to the enrolment exam.

BMI: body mass index, kg: kilograms.

### Objective (iii): Change in spirometry with age in BMI tertiles

**Figure 3.**
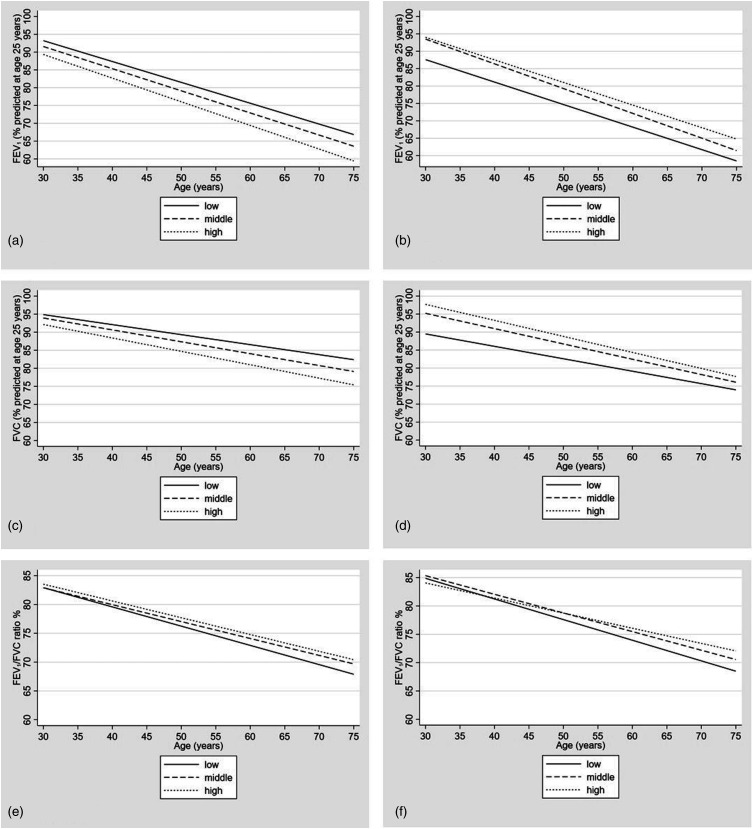
The change in FEV_1_, FVC and FEV_1_/FVC Ratio with age for tertiles of BMI (Figs a, c, e: Males. Figs b, d, f: Females). FEV_1_ and FVC are expressed as the percent predicted at age 25. The ratio of FEV_1_/FVC was calculated using the absolute values. FEV_1_: Forced Expiratory Volume in 1, FVC: Forced Vital Capacity, BMI: body mass index. Tertiles of BMI are based on individuals’ mean BMI below age 40 (low, middle and high tertiles).

One thousand four hundred forty-seven participants (656 men) were divided into tertiles (low, middle and high), characteristics for those who attended exam 2 are shown in Table S2. FEV_1_, FVC and FEV_1_/FVC ratio changes with age are shown in Figure 3. FEV_1_ and FVC decline was graphically steeper in middle and high tertiles than low, whilst FEV_1_/FVC ratio decline was graphically steeper in the low tertile.

### Objective (iv): Body composition, airflow obstruction and lung density

Adjusted logistic regression models for airflow obstruction and low lung density with body composition measures are provided in the supplement (Tables S3 and S4). Odds ratios for airflow obstruction development with lean mass index in men (0.90, Confidence Interval (CI): 0.67–1.20, *p* = 0.48), and women (0.85, CI: 0.65–1.12, *p* = 0.26) and with fat mass index in women (1.05, CI: 0.92–1.20, *p* = 0.49) were not significant. However, the odds ratio for airflow obstruction with fat mass index in men (0.71, CI: 0.51–0.98, *p* = 0.04) was significant. Odds ratios for low lung density with lean mass index in men (0.85, CI: 0.74–0.99, *p* = 0.03) and women (0.74, CI: 0.58–0.96, *p* = 0.02) were also significant, but not with fat mass index (men: 0.92, CI: 0.80–1.06, *p* = 0.24, women: 0.96, CI: 0.85–1.10, *p* = 0.57).

Visual differences observed in BMI between AO groups and non-AO smokers and between LLD groups and non-LLD smokers remained in earlier adulthood following adjustment with pack years in linear spline models (Figures S2 and S4 and Table S5). The separation remained in older women, but in men above 60 years AO participants had a higher BMI than non-AO smokers. When participants with a restrictive spirometry pattern were removed from the analysis the observed difference in predicted BMI between AO and non-AO and LLD and non-LLD groups was reduced (Figures S3 and S5, Table S5). Following adjustment with pack years and removal of participants with restrictive spirometry, the lowest BMI tertile in both sexes continued to have the steepest FEV_1_/FVC ratio decline on visual inspection (Figures S6 and S7).

## Discussion

Examination of longitudinal patterns of BMI in this large population-based cohort suggested a lower BMI throughout adulthood in participants who developed airflow obstruction during the study period compared to smokers and non-smokers who did not. Similarly, BMI was observed to be lower across adulthood in participants with low lung density at CT compared to smokers and non-smokers without low lung density. BMI between 35 and 45 years in linear spline models of BMI patterns across adulthood, was observed to be lower in AO than non-AO groups and in LLD than non-LLD groups. Comparison of age-related spirometry decline between BMI tertiles of subjects <40 years of age revealed that whilst subsequent decline in FEV_1_ and FVC appeared to be related to higher BMI, decline in FEV_1_/FVC ratio, the spirometric index of airflow obstruction, was graphically steepest in the lowest BMI tertile. These findings remained after adjustment for smoking pack years. The steeper FEV_1_/FVC ratio decline in the lowest BMI tertile and the lower BMI throughout adulthood in participants who developed airflow obstruction and/or low lung density supports the notion that earlier life body mass status may influence age-related impairment of lung function and structure. Further support for this hypothesis is provided by our concordant observation of associations between earlier life body mass and the development of low lung density on CT imaging. We acknowledge that absolute differences in measures of AO and Lung density between groups may be small and of uncertain clinical significance in an individual. However, the purpose of our analysis was to understand temporal relationships between indices of lung function and structure and measures of body habitus across populations in order to advance hypotheses about pathophysiology. We suggest results may be of pathophysiological significance in the development of COPD but recognise mechanistic links between body habitus and the development of lung damage will require prospective investigation.

Whilst the existence of a relationship between low BMI and COPD is known, the sequence and timing in the natural history of the disease is uncertain.^11,20,21^ Baseline differences in BMI between participants with obstructive lung disease and non-smoking controls was reported in a study examining decline in body mass, suggesting the association might have origins earlier in life.^10^ The risk of developing COPD in the Baltimore Longitudinal Study of Ageing was higher in men with a lower BMI, but incident cases in women were insufficient to assess associations.^22^ Previous prospective studies have demonstrated results consistent with our findings, but are limited by absence of spirometry and/or short duration of follow-up.^12,23^ Our observation that decline in FEV_1_/FVC ratio was steeper in the lowest BMI tertile and that higher BMI was related to greater decline in FEV_1_ and FVC is in line with analyses from the European Community Respiratory Health Survey (ECRHS).^13^ We advance previous investigations by examining associations longitudinally over a substantially longer period of adulthood and triangulating findings with measurements of lung density and body composition. We observed lower spirometric values (FEV_1_ and FVC) in the lowest BMI categories in women (Figure 3b and d) but the highest BMI categories in men (Figure 3a and c) which contrasts the findings from the ECRHS study^13^ but is in line with other previous reports.^24^ The explanation for these differences, and potential implication of fat distribution differences between men and women, requires further study.

A greater difference in predicted BMI in linear spline models occurred between groups based on lung density compared to spirometric measures of obstruction. This may reflect stronger associations between BMI and structural rather than functional changes, which would be in line with descriptions of a lower BMI in emphysematous phenotypes in COPD.^25,26^

The notion that body mass plays a role in the pathogenesis of COPD is supported by observations that starvation states (for example severely calorie restricted inhabitants of the WWII Warsaw Ghetto and anorexia nervosa) may prompt the development of emphysema-like changes in the lung.^27,28^ Rodent studies have observed emphysema-type changes in low energy states.^29^ We speculate that body mass, representing body tissue reserves may interact with an insult, such as smoking to increase susceptibility to the development of structural lung changes. Cross-sectional studies indicate that lean mass depletion in COPD is associated with the presence and extent of emphysema.^30^ We extend previous observations by investigating relationships between lean and fat mass and the longitudinal development of low lung density, demonstrating that higher lean mass was statistically associated with lower odds ratios for low lung density in both sexes. A statistically significant relationship between a higher odds ratio of airflow obstruction and lower fat mass, however, was found in men. The fact that the cohort studied contained BMIs mostly within normal and obese categories may have impacted the findings of relationships with body composition, but also raises questions about the optimal BMI for reduction of risk of airflow obstruction or low lung density as previously defined by the ‘obesity paradox’.^9^ The findings have particular significance where poor socioeconomic status is associated with both a low BMI and a high use of biomass fuels, where levels of COPD are increasing.^31^ Additional analyses suggested that the associations we observed were not fully accounted for by smoking volume. However, this does not rule out an interaction between smoke inhalation and body habitus at a mechanistic level.

There are limitations to the conclusions we can draw from these data. Pre-bronchodilator spirometry was used to determine the airflow obstruction group, which may therefore include participants with reversibility, although we note other cohort studies e.g., the Copenhagen city heart study have provided important insight into the natural history of lung function decline using pre-bronchodilator spirometry. The definition of AO based on a single occurrence may lack permanence, however we believe this is adequate for a population study and the distribution of the incidence of AO supports this with the highest frequency between ages of 60 and 70 years. Whilst we have adjusted for known confounders such as smoking, in our models, we cannot exclude the impact of other unmeasured confounders impacting the observations, a limitation present in all observational studies. We base our conclusions on observed differences between groups over time from visual inspection of spline curves. We cannot rule out the possibility that our findings occurred by chance but believe the concordance of our observations through triangulation of relationships with both AO and LLD and through our analysis of time-course changes between BMI categories makes this unlikely. The integration of evidence from different complementary analyses lends additional weight to our interpretation. Given that this was a large non-disease-based population we believe that our findings are potentially applicable to the development of lung pathophysiology in COPD.

It is possible that associations between lung function and structure and BMI have changed over time, but age-period-cohort effects were not specifically studied within the analysis. Confirmation of our results would clearly be desirable in lifelong cohorts of people who go on to develop significant COPD to more clearly determine the role of early life exposures in the results observed in our study.

## Conclusion

The graphs from BMI trajectory models across adulthood for groups based on outcomes of airflow obstruction or lung density together with the observation of a steeper decline in FEV_1_/FVC ratio suggest that lower earlier BMI may be associated with the development of airflow obstruction and low lung density. The relationships with BMI and functional and structural changes within the lung may in turn suggest a role for body mass in the development of COPD.

## Supplemental Material

Supplemental Material - Body mass index across adulthood and the development of airflow obstruction and emphysema Click here for additional data file. Supplemental Material for Body mass index across adulthood and the development of airflow obstruction and emphysema by Ruth E Trethewey, Nicole L Spartano, Ramachandran S Vasan, Martin G Larson, George T O’Connor, Dale W Esliger, Emily S Petherick and Michael C Steiner in Chronic Respiratory Disease

## References

[1] Schols AM Soeters PB Dingemans AM, et al. Prevalence and characteristics of nutritional depletion in patients with stable COPD eligible for pulmonary rehabilitation. Am Rev Respir Dis 1993; 147(5): 1151–1156. 8484624 10.1164/ajrccm/147.5.1151

[2] Prescott E Lange P Vestbo J. Socioeconomic status, lung function and admission to hospital for COPD: results from the Copenhagen City Heart Study. Eur Respir J 1999; 13(0903–1936): 1109–1114. 10414412 10.1034/j.1399-3003.1999.13e28.x

[3] Landbo C Prescott E Lange P, et al. Prognostic value of nutritional status in chronic obstructive pulmonary disease. Am J Respir Crit Care Med 1999; 160: 1856–1861. 10588597 10.1164/ajrccm.160.6.9902115

[4] Shoup R Dalsky G Warner S, et al. Body composition and health-related quality of life in patients with obstructive airways disease. Eur Respir J 1997; 10: 1576–1580. 9230250 10.1183/09031936.97.10071576

[5] Engelen MP Schols AM Baken WC, et al. Nutritional depletion in relation to respiratory and peripheral skeletal muscle function in out-patients with COPD. Eur Respir J 1994; 7(10): 1793–1797. 7828687 10.1183/09031936.94.07101793

[6] Palange P Forte S Felli A, et al. Nutritional state and exercise tolerance in patients with COPD. Chest 1995; 107: 1206–1212. 7750307 10.1378/chest.107.5.1206

[7] Vestbo J Prescott E Almdal T, et al. Body mass, fat-free body mass, and prognosis in patients with chronic obstructive pulmonary disease from a random population sample: findings from the Copenhagen City Heart Study. Am J Respir Crit Care Med 2006; 173: 79–83. 16368793 10.1164/rccm.200506-969OC

[8] Maltais F Decramer M Casaburi R, et al. An official American thoracic society/European respiratory society statement: update on limb muscle dysfunction in chronic obstructive pulmonary disease. Am J Respir Crit Care Med 2014; 189(9): e15–e62. 24787074 10.1164/rccm.201402-0373ST PMC4098112

[9] Schols A Ferreira I Franssen F, et al. An official European respiratory society statement. Nutritional assessment and therapy in COPD. Eur Respir J 2014; 44(6): 1504–1520. 25234804 10.1183/09031936.00070914

[10] van den Borst B Koster A Yu B, et al. Is age-related decline in lean mass and physical function accelerated by obstructive lung disease or smoking? Thorax 2011; 66: 961–969. 21724748 10.1136/thoraxjnl-2011-200010 PMC3285455

[11] Guerra S Sherrill DL Bobadilla A, et al. The relation of body mass index to asthma, chronic bronchitis, and emphysema. Chest 2002; 122(4): 1256–1263. 12377850 10.1378/chest.122.4.1256

[12] Behrens G Matthews CE Moore SC, et al. Body size and physical activity in relation to incidence of chronic obstructive pulmonary disease. CMAJ 2014; 186(12): E457–E469. 25002559 10.1503/cmaj.140025 PMC4150732

[13] Peralta GP Marcon A Carsin AE, et al. Body mass index and weight change are associated with adult lung function trajectories: the prospective ECRHS study. Thorax 2020; 75(4): 313–320. 32098862 10.1136/thoraxjnl-2019-213880 PMC7231449

[14] Thyagarajan B Jacobs DR Apostol GG, et al. Longitudinal association of body mass index with lung function: the CARDIA study. Respir Res 2008; 9: 31. 18394165 10.1186/1465-9921-9-31 PMC2386787

[15] Lawlor DA Tilling K Davey Smith G. Triangulation in aetiological epidemiology. Int J Epidemiol 2016; 45(6): 1866–1886. 28108528 10.1093/ije/dyw314 PMC5841843

[16] Kannel WB Feinleib M McNamara PM, et al. An investigation of coronary heart disease in families. The Framingham offspring study. Am J Epidemiol 1979; 110(3): 281–290. 474565 10.1093/oxfordjournals.aje.a112813

[17] Quanjer PH Tammeling GJ Cotes JE, et al. Lung volumes and forced ventilatory flows. Report Working Party Standardization of Lung Function Tests, European Community for Steel and Coal. Official Statement of the European Respiratory Society. Eur Respir J Suppl 1993; 16: 5–40. 8499054

[18] Tsao CW Vasan RS. Cohort Profile: The Framingham Heart Study (FHS): overview of milestones in cardiovascular epidemiology. Int J Epidemiol 2015; 44(6): 1800–1813. 26705418 10.1093/ije/dyv337 PMC5156338

[19] Leckie G Charlton C. runmlwin: a program to run the MLwiN multilevel modeling software from within Stata. J Stat Softw 2013; 52: 1–40.23761062

[20] Gray-Donald K Gibbons L Shapiro SH, et al. Nutritional status and mortality in chronic obstructive pulmonary disease. Am J Respir Crit Care Med 1996; 153(3): 961–966. 8630580 10.1164/ajrccm.153.3.8630580

[21] Vermeeren MA Creutzberg EC Schols AM, et al. Prevalence of nutritional depletion in a large out-patient population of patients with COPD. Respir Med 2006; 100(8): 1349–1355. 16412624 10.1016/j.rmed.2005.11.023

[22] Harik-Khan RI Fleg JL Wise RA. Body mass index and the risk of COPD. Chest 2002; 121(2): 370–376. 11834645 10.1378/chest.121.2.370

[23] Zhou Y Wang D Liu S, et al. The association between BMI and COPD: the results of two population-based studies in Guangzhou, China. COPD 2013; 10(5): 567–572. 23844907 10.3109/15412555.2013.781579

[24] Harik-Khan RI Wise RA Fleg JL. The effect of gender on the relationship between body fat distribution and lung function. J Clin Epidemiol 2001; 54(4): 399–406. 11297889 10.1016/s0895-4356(00)00318-8

[25] Ogawa E Nakano Y Ohara T, et al. Body mass index in male patients with COPD: correlation with low attenuation areas on CT. Thorax 2009; 64(1): 20–25. 18852156 10.1136/thx.2008.097543

[26] Kitaguchi Y Fujimoto K Kubo K, et al. Characteristics of COPD phenotypes classified according to the findings of HRCT. Respir Med 2006; 100(10): 1742–1752. 16549342 10.1016/j.rmed.2006.02.003

[27] Winick M. Hunger Disease. Studies by the Jewish Physician in the Warsaw Ghetto*.* New York, NY: John Wiley & Sons; 1979.

[28] Coxson HO Chan IH Mayo JR, et al. Early emphysema in patients with anorexia nervosa. Am J Respir Crit Care Med 2004; 170(7): 748–752. 15256394 10.1164/rccm.200405-651OC

[29] Massaro D Massaro GD. Hunger disease and pulmonary alveoli. Am J Respir Crit Care Med 2004; 170(7): 723–724. 15447950 10.1164/rccm.2408002

[30] Rutten EP Grydeland TB Pillai SG, et al. Quantitative CT: associations between emphysema, airway wall thickness and body composition in COPD. Pulm Med 2011; 2011: 419328. 21647214 10.1155/2011/419328 PMC3100107

[31] Hu G Zhou Y Tian J, et al. Risk of COPD from exposure to biomass smoke: a metaanalysis. Chest 2010; 138(1): 20–31. 20139228 10.1378/chest.08-2114

