# Supplementary material for: Body mass index across adulthood and the development of airflow obstruction and emphysema
Source: Chron Respir Dis. 2022 Nov 9;19:14799731221139294. doi: 10.1177/14799731221139294 (PMC9661572; doi:10.1177/14799731221139294)
Supplement: Supplemental Material - Body mass index across adulthood and the development of airflow obstruction and emphysema [file sj-pdf-1-crd-10.1177_14799731221139294.pdf]

Supplementary Material

Table of Contents:

Contents

Measurements 3

Inclusion Criteria 4

Statistical analysis: 5

(i) and (ii) Examination of BMI across the adult life span for airflow obstruction and non-airflow obstruction groups and low lung density and normal lung density groups: 5

(iii) Analysing the change in spirometry indices with age between BMI tertiles: 6

(iv) Assessment of the relationships between body composition with outcomes of airflow obstruction and low lung density: 7

Sensitivity analysis with adjustment for smoking volume using smoking pack years history 8

Results: 9

Distribution of Incidence of Airflow Obstruction across examination cycles and ages for the cohort period 9

Figure S1: Bar chart showing the frequency of participants classified as having airflow obstruction for different age categories over the cohort period 9

Best fitting fractional polynomial transformations of age for BMI trajectories 9

Table S1: Predicted values for BMI for the groups that developed and did not develop airflow obstruction and low lung density using spline models at age 35 and age 45 years 10

Table S2: Characteristics for the tertiles of BMI at Exam 2 11

Table S3: Adjusted logistic regression models for the presence of airflow obstruction with body composition measurements 12

Table S4: Adjusted logistic regression models for the presence of low lung density with body composition measures 13

Table S5: Predicted values for BMI at age 35 years and age 45 years for groups that developed and did not develop airflow obstruction and low lung density using spline models with pack years and participants who developed restrictive spirometry removed 14

Figure S2: Trajectories for BMI with age in those who develop airflow obstruction and smokers who do not adjusted for pack years. a. men, b. women 15

Figure S3: Trajectories for BMI with age in those who develop airflow obstruction and smokers and non-smokers who do not adjusted for pack years with participants who develop restrictive pattern removed. a. men, b. women 16

Figure S4: Trajectories for BMI with age in those with low lung density at CT and smokers without adjusted for pack years a. men, b. women 17

Figure S5: Trajectories for BMI with age in those with low lung density at CT and smokers without adjusted for pack years with participants who develop restrictive pattern removed. a. men, b. women 18

Figure S6: The change in FEV_1/FVC Ratio with age for BMI adjusted for pack years. a. men, b. women 20

Figure S7: The change in FEV_1/FVC Ratio with age for BMI adjusted for pack years with participants who develop a restrictive pattern removed. a. men b. women 21

References 22

Measurements

Spirometry

Lung function was assessed by spirometry, performed at Exams 2, 3 and 5-9 using a 6L Collins water-sealed bell spirometer with participants in the standing position. The procedure was repeated until three acceptable spirograms were achieved, up to a maximum of eight times as previously described, and the best readings of values including FEV_1 and FVC were recorded.[1] Post-bronchodilator recordings were not included at all examinations, so only pre-bronchodilator values were used, reversibility of airflow obstruction was therefore not incorporated into definitions.

BMI

BMI was calculated from weight (in kilograms)/height (in metres) squared, which were measured at each examination by a trained researcher, as weight/height squared.

CT

A subsample of 1368 participants (42% male, mean age 64.2 years) underwent a chest CT scan between 2002 and 2005, between Exams 7 and 8. Quantitative analysis of the lungs was performed on images acquired using an eight-slice multidetector CT at full inspiration in supine position (Lightspeed Ultra, GE, Milwaukee, WI, USA). Objective lung measures for lung volume and density have been produced through software based on 3D Slicer (www.Slicer.org). These measures include the fraction of lung below -950 Hounsfield Units.

DEXA Scans

Participants who attended Exam 6 or 7 were invited to attend a DEXA scan. These took place between 1995 and 2001 and were performed using a Lunar Dual X-ray absorptiometer (DPX-L) in the "fast" mode. The scans provided measures of whole-body fat mass and fat-free mass. Lean mass was

calculated as fat-free mass minus bone mineral density.[2] The indices of fat mass and lean mass were used to adjust for height, and were calculated as fat mass and lean mass divided by height (in metres) squared.

Inclusion Criteria

For objective (i) participants who had both valid spirometry and BMI measurements recorded between exams 2 and 9 were eligible for inclusion. They were also required to be >25 years of age, therefore some participants became eligible at later exams. Participants with airflow obstruction at any point below age 35 years or who only contributed measurements with an average age of 30 years or below were excluded to reduce the possibility of airflow obstruction preceding weight loss and to avoid bias from a lack of longitudinal data.

For objective (ii) participants were included if they underwent lung CT and had valid BMI measurements recorded between exams 2 and 9. Data was only used where age was ≥25 years.

For objective (iii) participants who had longitudinal data that included valid spirometry and BMI measurements between Exams 2 and 9, with at least one measurement below age 40 years were included. Only data from participants over 25 years of age was included therefore participants may not have been eligible at all exams.

For objective (iv) participants were included if they underwent DEXA (performed between 1995 and 2001) and had valid spirometry measures at Exam 8 or had lung CT measures recorded between 2002 and 2005.

Statistical analysis:

(i) and (ii) Examination of BMI across the adult life span for airflow obstruction and non-airflow obstruction groups and low lung density and normal lung density groups:

Statistical analysis for Objectives (i) and (ii) was undertaken in two phases. Firstly, fractional polynomial models were used for initial modelling of BMI trajectories in airflow obstruction groups and lung density groups. Subsequently, piecewise linear spline models were performed to allow direct comparison of predicted BMI at specific ages. These analyses enable comparison between unmatched groups across different ages and overcomes issues such as collinearity of repeated measures and missing data, therefore maximising the number of observations contributing to the analysis.[3] The fractional polynomial transformation of age, described by Royston and Altman, involves the use of power functions or a combination of two power functions of age, thereby allowing the selection of a variety of curve shapes.[4] Participants who only contributed data with a mean age below 30 years and those who had airflow obstruction below the age of 35 years at Exam 2 (n=44) were excluded so that inferences about the temporal relationship between BMI and airflow obstruction at these earlier ages might be made. Subjects over the age of 35 with airflow obstruction at study enrolment are included in the analysis as their data contributes at the appropriate age to the overall trajectory. Apart from the aforementioned exclusion all observations from participants between Exam 2 and Exam 9 with valid BMI, spirometry and, where relevant CT data, were included in the analysis. The fractional polynomial models for BMI change with age for each group (AO, Non-AO-S and Non-AO-N and LLD,NLD-S and NLD-N) were performed with the Exam at level 1 and the individual at level 2 with each of the standard powers (-2, -1, -0.5, 0, 0.5, 1, 2, 3, where power 0 is the log function) and combination of pairs of these powers for age.[5] Best fitting models were

selected based on the deviance from each model using the iterative generalised least squares (IGLS) estimation algorithm. Knot points for piecewise linear spline models were chosen based on visual inspection of the fractional polynomial models, as previously described.[5] The linear nature of the spline models allows the predicted BMI from the models at specific ages to be compared.

We undertook a stratified analysis with the objective of investigating trajectories of change within pre-specified AO/LD groups as a direct statistical comparison of the models is not possible. Conclusions from these analyses were drawn from visual inspection of the models.

(iii) Analysing the change in spirometry indices with age between BMI tertiles:

In a separate analysis, changes in FEV_1 (forced expiratory volume in 1 second), FVC (forced vital capacity) and FEV_1/FVC ratio with age were compared between different BMI tertiles (low, middle and high) to assess whether lower BMI was associated with faster lung function decline. BMI tertiles were chosen due to the unequal distribution of participants between standard BMI categories. To standardise values the percent of age 25 predicted ("%age25") FEV_1 and FVC values were used in the analysis of change in spirometry measures. These were calculated as the measured value divided by the predicted value for the individual at age 25 years (i.e. predicted peak lung function) using the Global Lung Initiative reference value presented as a percentage. The ratio of FEV_1/FVC was calculated using the absolute values. For the purpose of these calculations ethnicity was assumed to be "white" in view of its predominance within the cohort.[6] Height was adjusted for within the analysis and men and women modelled separately in view of differences in spirometry decline between sexes.

The shape of the change in %age25FEV_1, %age25FVC and ratio of FEV_1/FVC trajectories with age for all participants was first examined by fitting both fractional polynomial and linear models. The shape of the best fitting models for the trajectories was a linear slope beyond the age of 30, so multilevel

linear regression analysis was used to model the change in FEV_1, FVC and the ratio with age. This was done separately for different BMI categories. Participants who had both body mass and spirometry data available below the age of 40 years and subsequently were included in this analysis. The BMI tertiles were determined by the subject's mean BMI at examinations that took place under the age of 40 years (i.e. prior to the majority of COPD diagnoses). Smoking status was included as a single time point individual covariate.

(iv) Assessment of the relationships between body composition with outcomes of airflow obstruction and low lung density:

Participants who attended Exam 6 or 7 were invited to attend a DEXA scan. These took place between 1995 and 2001 and were performed using a Lunar Dual X-ray absorptiometer (DPX-L) in the "fast" mode. The scans provided measures of whole-body fat mass and fat-free mass. Lean mass was calculated as fat-free mass - bone mineral density.[2] The relationship between lean mass index and fat mass index at DEXA with the outcome of firstly the presence of airflow obstruction at Exam 8 (2005 to 2008) and secondly low lung density on CT (performed between 2005 and 2008) was assessed using logistic regression.

Inclusion criteria:

Participants were eligible for inclusion if they underwent a DEXA scan and had valid spirometry measures for outcomes of the presence or absence of airflow obstruction, or had undergone lung CT for outcomes of the presence or absence of low lung density. The indices of fat mass and lean mass were used to adjust for height and calculated as fat mass and lean mass divided by $height^2$. Those with airflow obstruction at Exam 6, which was close in time to when the DEXA scans were performed, were excluded from this analysis. The adjusted models were analysed separately for

each sex and included age and pack years with mean centred values for lean mass index, fat mass index and age.

Sensitivity analysis with adjustment for smoking volume using smoking pack years history

A sensitivity analysis was undertaken with the inclusion of smoking pack years data for airflow/non airflow obstruction smokers and for low lung density and normal lung density smokers to examine whether any differences between these groups found in previous models remains after the adjustment for the volume of smoking. There were 4144 participants (M=2001) with data available for the sub-analysis based on presence/absence of airflow obstruction outcomes for the BMI trajectory models across adulthood. This was done using linear spline models with the previously determined knot points and the inclusion of pack years as a continuous level 2 covariate; sample size was reduced because pack years information was not universally available. In view of a potential relationship between a restrictive spirometry pattern and increasing BMI, which might appear to increase the BMI in non-airflow obstruction participants, the sub-analysis was also repeated with participants who developed an FEV_1<80 together with an FVC<80 removed (411 participants in total removed for AO/non-AO models. A sub-analysis with pack years included as a covariate was also performed for the linear models for the FEV_1/FVC ratio decline with age for the BMI tertiles, in 1272 (m=602) participants in whom the data was available, to examine whether smoking volume affected differences in changes in lung function between tertiles of BMI. This sub-analysis was also repeated with participants who developed a restrictive pattern of spirometry removed. The results of these analyses can be found in Figures S2 – S7.

Results:

Distribution of Incidence of Airflow Obstruction across examination cycles and ages for the cohort period

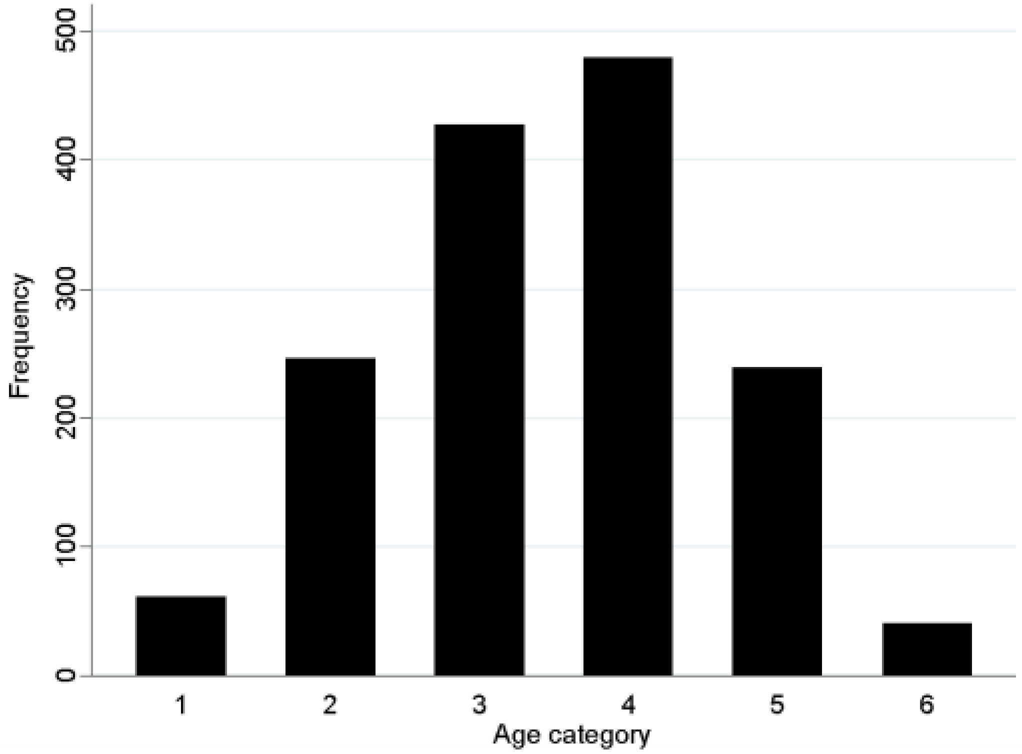

Figure S1: Bar chart showing the frequency of participants classified as having airflow obstruction for different age categories over the cohort period

Age categories: 1: 35-40 years, 2: 40-50 years, 3: 50-60 years, 4: 60-70 years, 5: 70-80 years, 6: >80 years

Best fitting fractional polynomial transformations of age for BMI trajectories

The best fitting fractional polynomial transformations of age for BMI change with age for men were the pair of powers 0, -1 for non-AO-N, 2, 1 for non-AO-S and 2, 1 for AO (figure 2a). For women (figure 2b) they were 2, 2 for non-AO-N, 3, 2 for non-AO-S and 3, 3 for AO.

The best fitting fractional polynomials for the pattern of BMI change with age in men were the pair of powers 2, 2 for NLD-N, 2, 1 for NLD-S and 2, 0.5 for LLD. In women, they were 2, 2 for NLD-N, 3, 2 for NLD-S and 3, 3 for LLD.

Table S1: Predicted values for BMI for the groups that developed and did not develop airflow obstruction and low lung density using spline models at age 35 and age 45 years

	**Predicted BMI age 35 years**		**Predicted BMI age 45 years**	
	(BMI – kg/m^2)			
	**Men**	**Women**	**Men**	**Women**
People with airflow obstruction (AO)	24.7	21.8	26.7	24.6
Ever-smokers non-airflow obstruction (non-AO-S)	25.3	23.0	27.1	25.5
Never-smokers non-airflow obstruction (non-AO-N)	25.9	22.9	27.3	25.5
People with low lung density (LLD)	24.5	21.5	26.4	23.4
Ever-smokers normal lung density (NLD-S)	26.0	22.8	27.6	25.4
Never-smokers normal lung density (NLD-N)	25.7	22.9	27.6	25.1

Abbreviations: BMI: body mass index, m: metres, kg: kilograms

Ages 35 and 45 years selected as appropriate representation.

Predicted values are taken from linear spline models based on data from 4587 (AO vs non-AO groups) and 1368 (LLD vs non-LLD groups) subjects.

Table S2: Characteristics for the tertiles of BMI at Exam 2

	Men			Women		
	**Low** n=153	**Middle** n=155	**High** n=164	**Low** n=206	**Middle** n=196	**High** n=186
			Mean (SD)			
Age (years)	33.5 (4.9)	34.0 (4.7)	34.3 (4.6)	34.2 (4.0)	34.3 (4.3)	34.0 (4.5)
BMI (kg/m^2)	22.6 (1.5) *	25.7 (1.2) *	29.9 (3.1) *	19.8 (1.2) *	22.3 (1.1) *	28.0 (5.0) *
FEV_1 (%predicted age 25)	84.5 (22.9)	85.8 (19.6)	83.1 (20.8)	80.1 (26.2)	85.8 (23.7)	80.8 (25.5)
FVC (% predicted age 25)	87.4 (22.9)	88.4 (18.9)	84.0 (21.0)	82.3 (26.1)	88.6 (23.9)	82.9 (26.1)
FEV_1/FVC	81.1 (7.9)	81.4 (7.9)	83.1 (6.2)	83.9 (8.4)	83.8 (6.8)	84.1 (6.8)
Smoking status - N^o smokers (%)	109 (71.2)	106 (68.4)	127 (77.4)	147 (71.4)	145 (74.0)	130 (69.9)
spirometric airflow obstruction - N^o (%)	46 (21.3)	43 (19.2)	37 (16.6)	56 (21.0)	53 (19.9)	49 (18.3)
Average N^o Pack years	8.4 (10.7)	8.6 (11.1)	10.2 (12.3)	7.22 (10.3)	8.49 (11.3)	7.8 (9.8)

Data shown for subjects attending exam 2 only (i.e. 1060 out of 1272)

Abbreviations: n: number of participants, SD: standard deviation, BMI: body mass index, kg: kilograms, m: metres, FEV_1: Forced Expiratory Volume in 1 second, FVC: Forced Vital Capacity, N^o: number, %: percentage of. Tertiles of BMI are based on individuals' mean BMI below age 40. [†]Pack year data only available for 885 subjects in this analysis. *Denotes statistical difference between tertiles

Table S3: Adjusted logistic regression models for the presence of airflow obstruction with body composition measurements

Model 1 with Lean mass

		Odds Ratio (CI)	P value
**Men** (n=442)			
	Lean mass index	0.90 (0.67 – 1.20)	0.48
	Pack years	1.05 (1.02 – 1.07)	<0.01
	Age	0.94 (0.87 – 1.02)	0.142
**Women** (n=760)			
	Lean mass index	0.85 (0.65 – 1.12)	0.26
	Pack years	1.03 (1.00 – 1.05)	0.02
	Age	1.02 (0.96 – 1.07)	0.60

Model 2 with Fat mass

**Men** (n=442)			
	Fat mass index	0.71 (0.51 – 0.98)	0.04
	Pack years	1.05 (1.03 – 1.08)	<0.01
	Age	0.95 (0.88 – 1.02)	1.07
**Women** (n=760)			
	Fat mass index	1.05 (0.92 – 1.20)	0.49
	Pack years	1.03 (1.00 – 1.05)	0.02
	Age	1.01 (0.96 – 1.07)	0.63

Abbreviations: n: number of participants, CI: confidence interval, CT: computed tomography. Fat-free mass, fat mass and age are mean centred.

Table S4: Adjusted logistic regression models for the presence of low lung density with body composition measures

Model 1 with Lean mass

		Odds Ratio (CI)	P value
**Men** (n = 284)			
	Lean mass index	0.85 (0.74 – 0.99)	0.03
	Pack years	1.02 (1.00 – 1.03)	0.02
	Age at CT	1.01 (0.98 – 1.04)	0.67
**Women** (n=456)			
	Lean mass index	0.74 (0.58 – 0.96)	0.02
	Pack years	1.02 (1.00 – 1.04)	0.01
	Age at CT	1.09 (1.04 – 1.15)	<0.01

Model 2 with Fat mass

		Odds Ratio (CI)	P value
**Men** (n=284)			
	Fat mass index	0.92 (0.80 – 1.06)	0.24
	Pack years	1.02 (1.00 – 1.03)	0.02
	Age at CT	1.01 (0.98 – 1.04)	0.64
**Women** (n=456)			
	Fat mass index	0.96 (0.85 – 1.10)	0.57
	Pack years	1.02 (1.00 – 1.04)	0.01
	Age at CT	1.08 (1.03 – 1.14)	<0.01

Abbreviations: n: number of participants, CI: confidence interval, CT: computed tomography. Fat-free mass, fat mass and age are mean centred.

Sensitivity analysis adjusting for pack years of smoking and the development of a restrictive pattern of spirometry

Table S5: Predicted values for BMI at age 35 years and age 45 years for groups that developed and did not develop airflow obstruction and low lung density using spline models with pack years and participants who developed restrictive spirometry removed

	**Predicted BMI age 35y**		**Predicted BMI age 45y**	
	(BMI – kg/m^2)			
	**Men**	**Women**	**Men**	**Women**
**Models including pack years adjustment**				
Airflow obstruction (AO)	24.6	21.5	26.6	24.4
Ever-smokers non-airflow obstruction (non-AO-S)	25.3	22.8	27.1	25.3
Low lung density (LLD)	24.4	20.9	26.2	23.5
Ever-smokers normal lung density (NLD-S)	25.9	22.7	27.6	25.4
**Models including pack years adjustment and participants with restrictive pattern on spirometry removed**				
Airflow obstruction (AO)	24.4	21.0	26.3	24.1
Ever-smokers non-airflow obstruction (non-AO-S)	25.2	22.9	27.1	25.4
Never-smokers non-airflow obstruction (non-AO-N)	25.9	22.9	27.3	25.4
Low lung density (LLD)	24.5	20.8	26.2	23.4
Ever-smokers normal lung density (NLD-S)	25.5	22.7	27.3	25.2
Never-smokers normal lung density (NLD-N)	25.5	22.9	27.4	25.0

Abbreviations: y: years, BMI: body mass index, m: metres, kg: kilograms, n (number of participants)

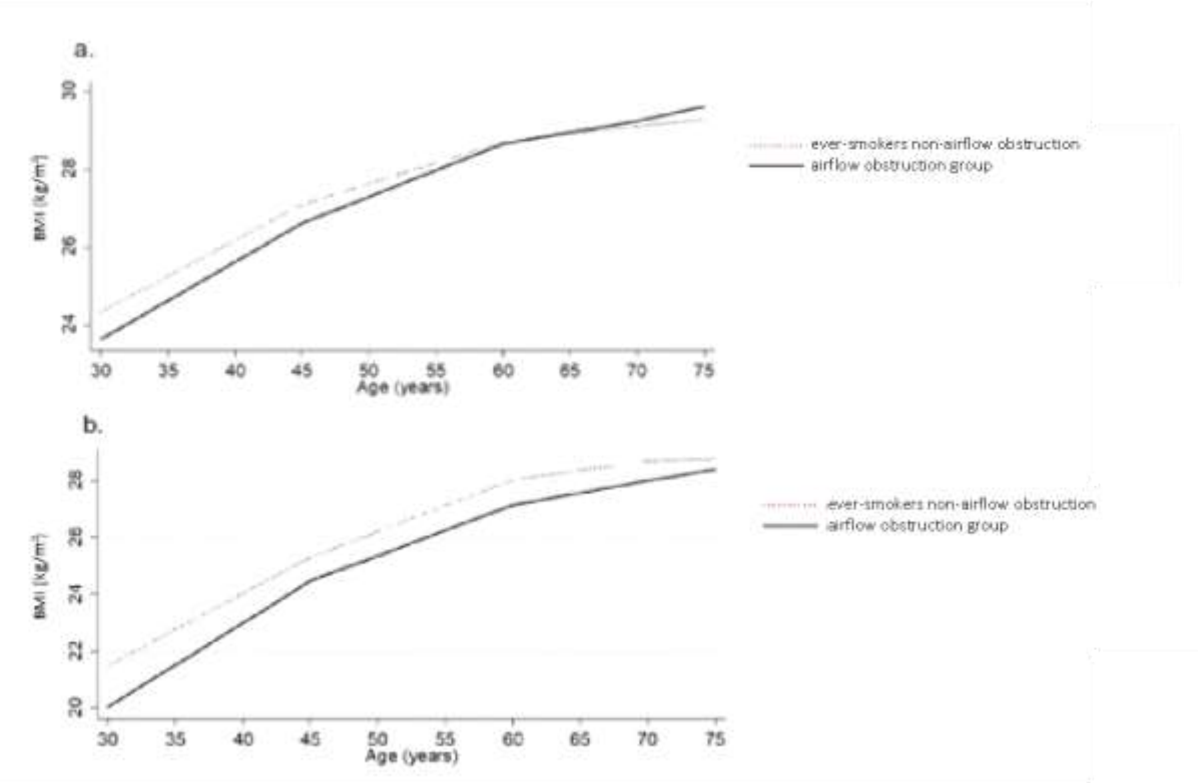

Figure S2: Trajectories for BMI with age in those who develop airflow obstruction and smokers who do not adjusted for pack years. a. men, b. women

Abbreviations: BMI: body mass index, kg: kilograms, m: metre.

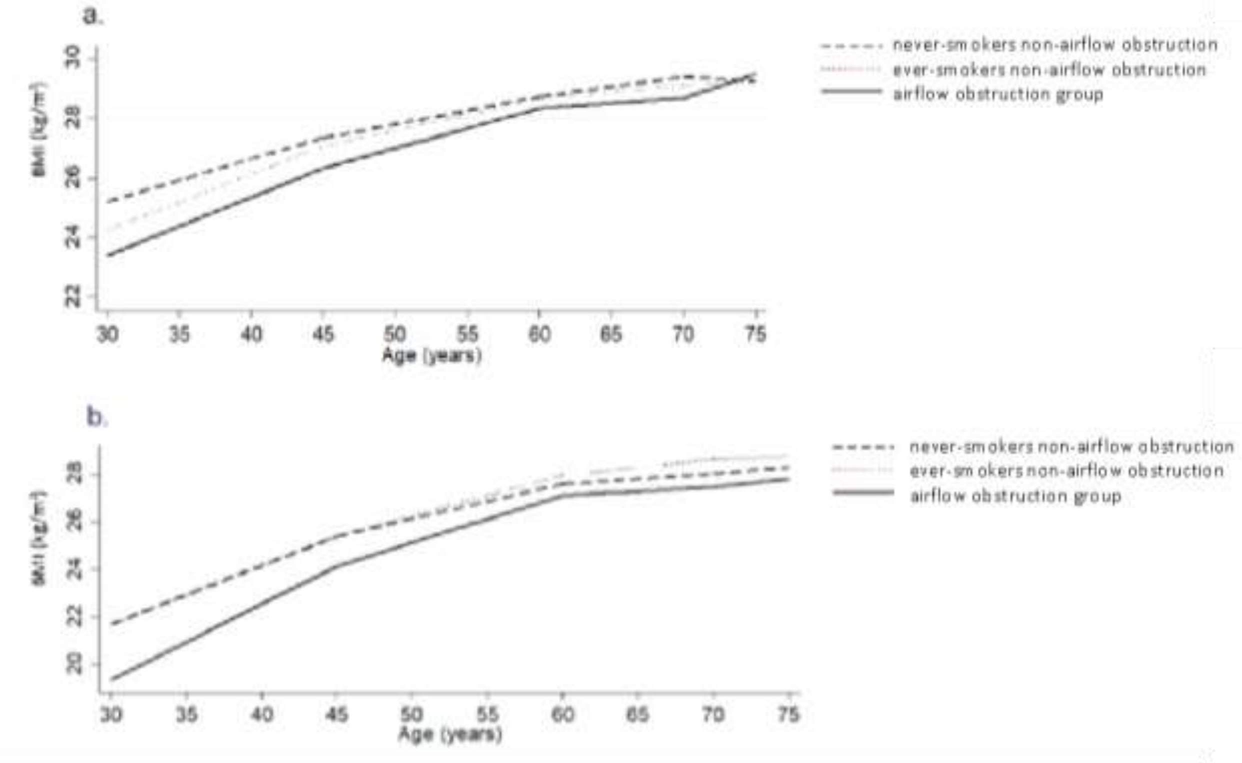

Figure S3: Trajectories for BMI with age in those who develop airflow obstruction and smokers and non-smokers who do not adjusted for pack years with participants who develop restrictive pattern removed. a. men, b. women

Abbreviations: BMI: body mass index, kg: kilograms, m: metre.

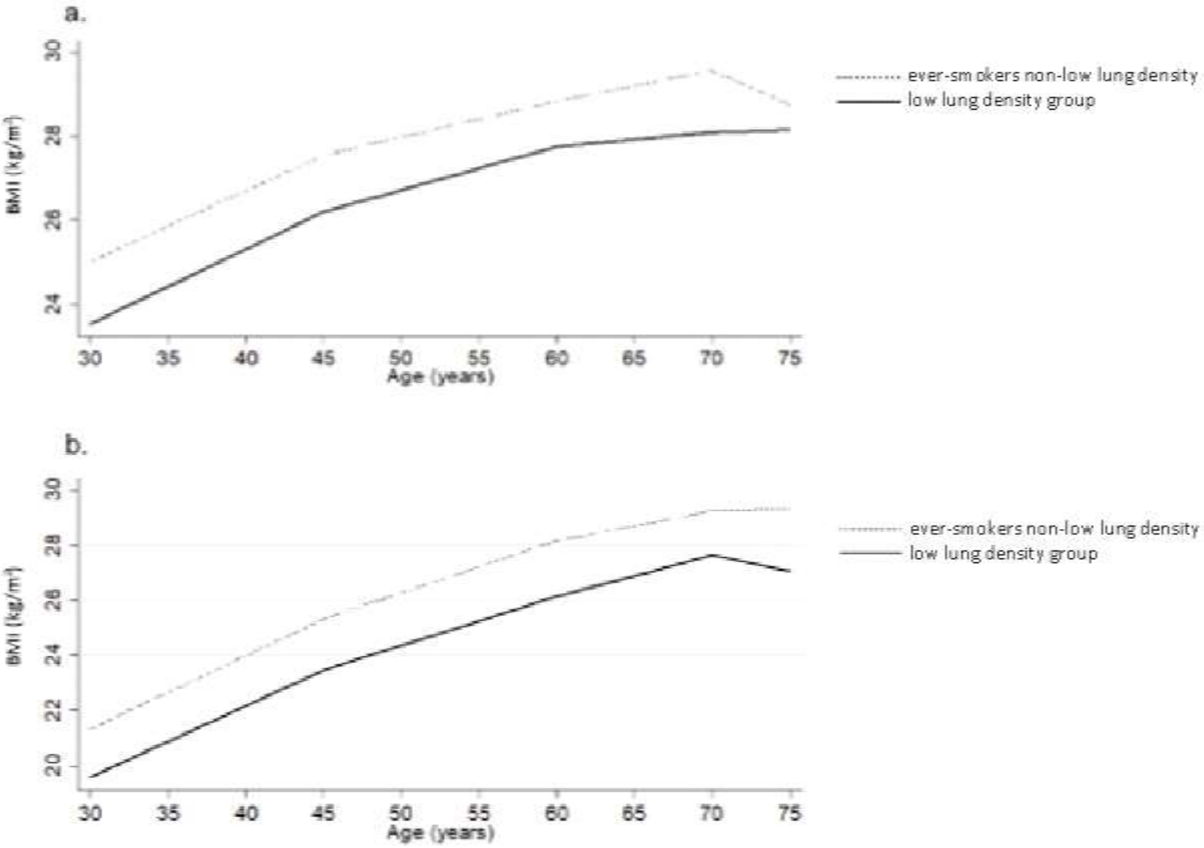

Figure S4: Trajectories for BMI with age in those with low lung density at CT and smokers without adjusted for pack years a. men, b. women

Abbreviations: BMI: body mass index, kg: kilograms, m: metre.

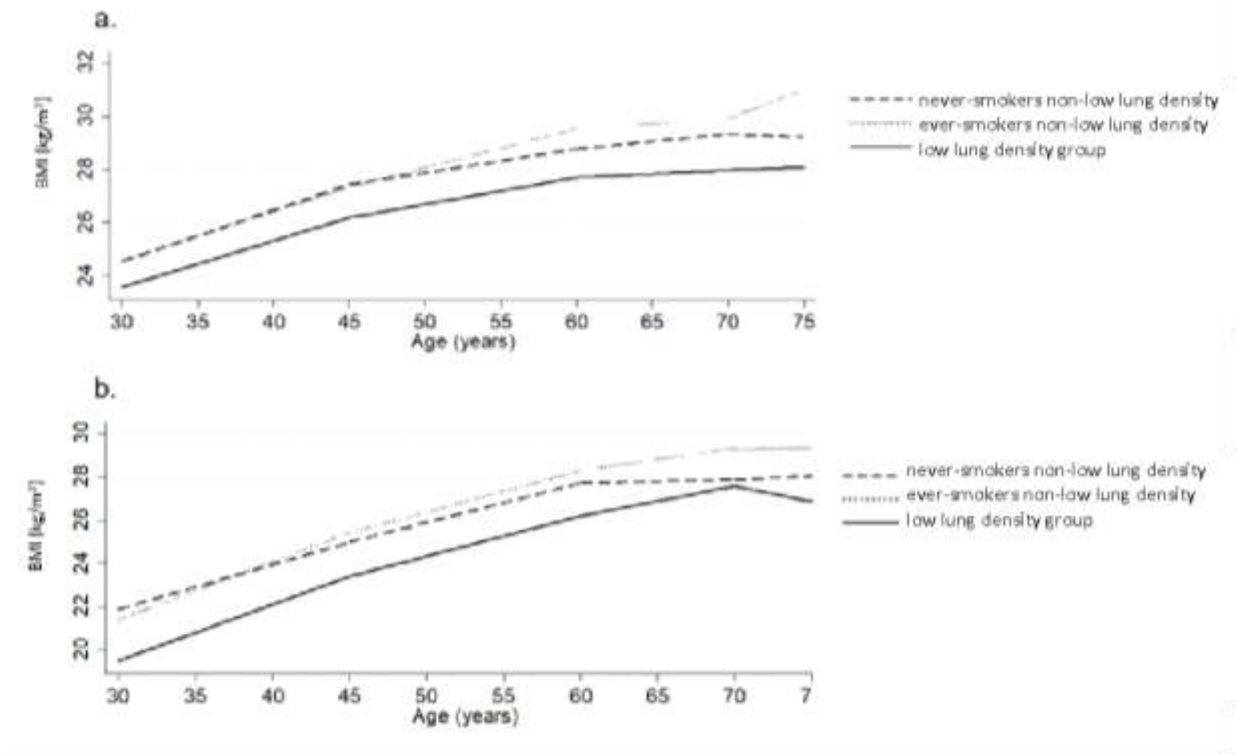

Figure S5: Trajectories for BMI with age in those with low lung density at CT and smokers without adjusted for pack years with participants who develop restrictive pattern removed. a. men, b. women

Abbreviations: BMI: body mass index, kg: kilograms, m: metre.

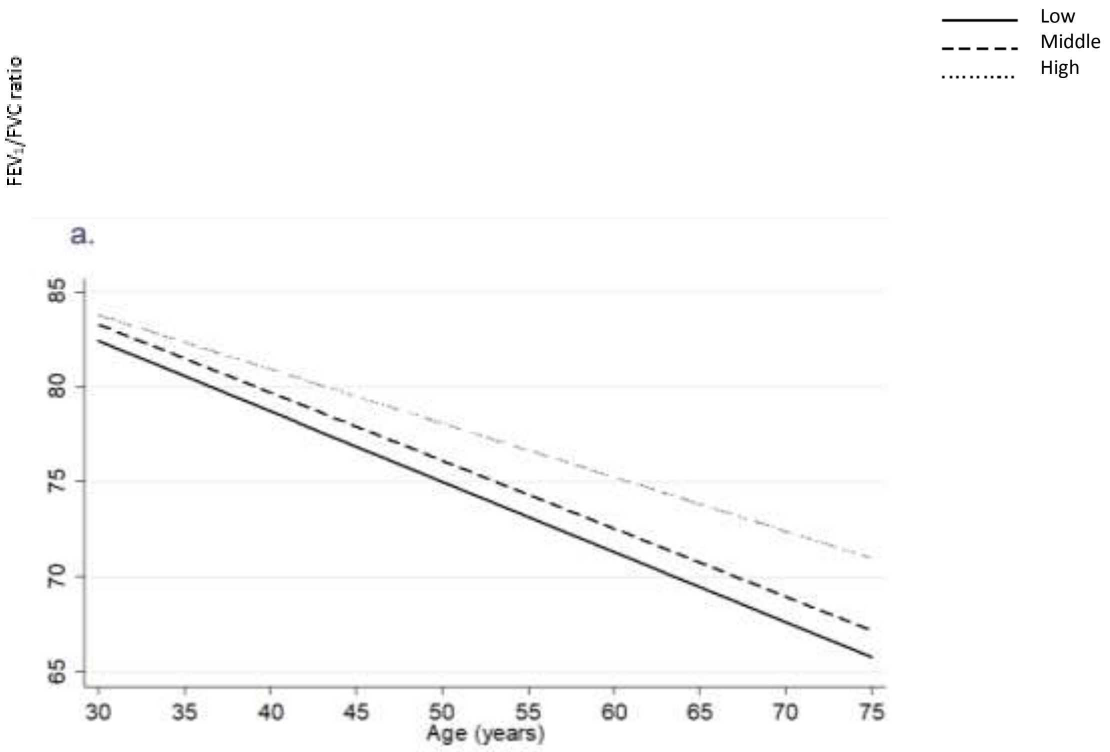
Low
Middle
High
FEV1/FVC ratio
a.
85
80
75
70
65
30
35
40
45
50
55
60
65
70
75
Age (years)

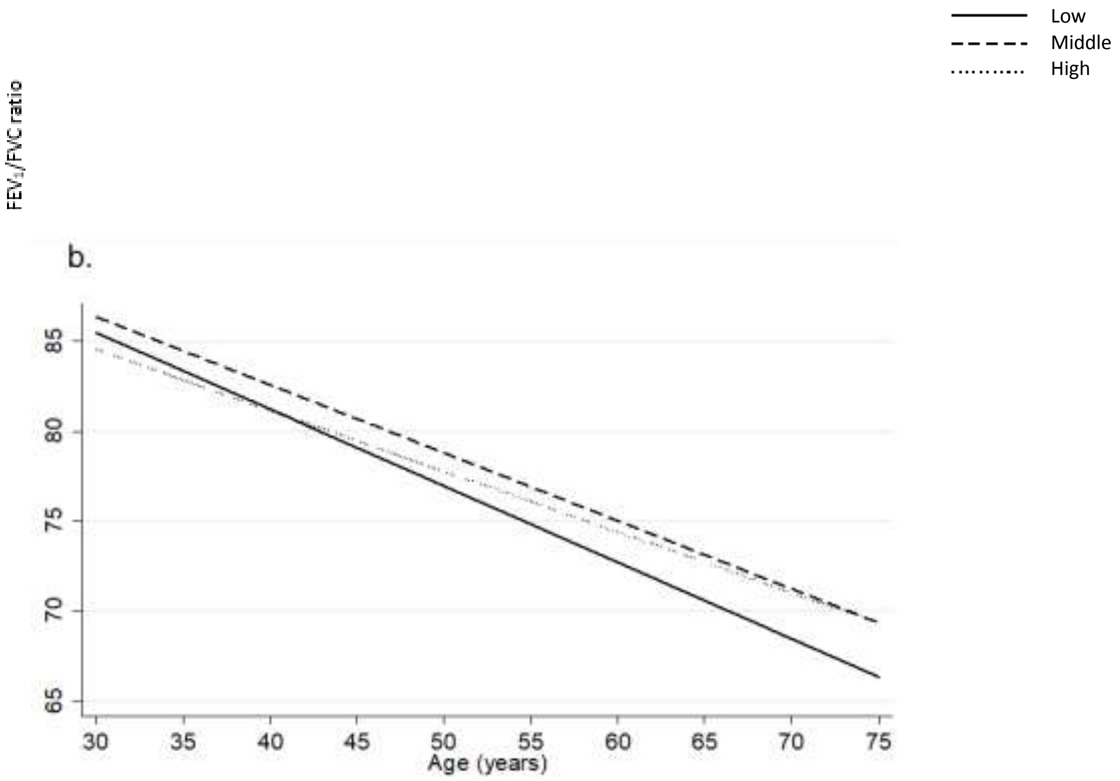
Low
Middle
High
FEV1/FVC ratio
b.
85
80
75
70
65
30
35
40
45
50
55
60
65
70
75
Age (years)

Figure S6: The change in FEV_1/FVC Ratio with age for BMI adjusted for pack years. a. men, b. women

Abbreviations: FEV_1/FVC: ratio of the Forced Expiratory Volume in 1 second/Forced Vital Capacity, BMI: body mass index. Tertiles of BMI are based on individuals' mean BMI below age 40 years.

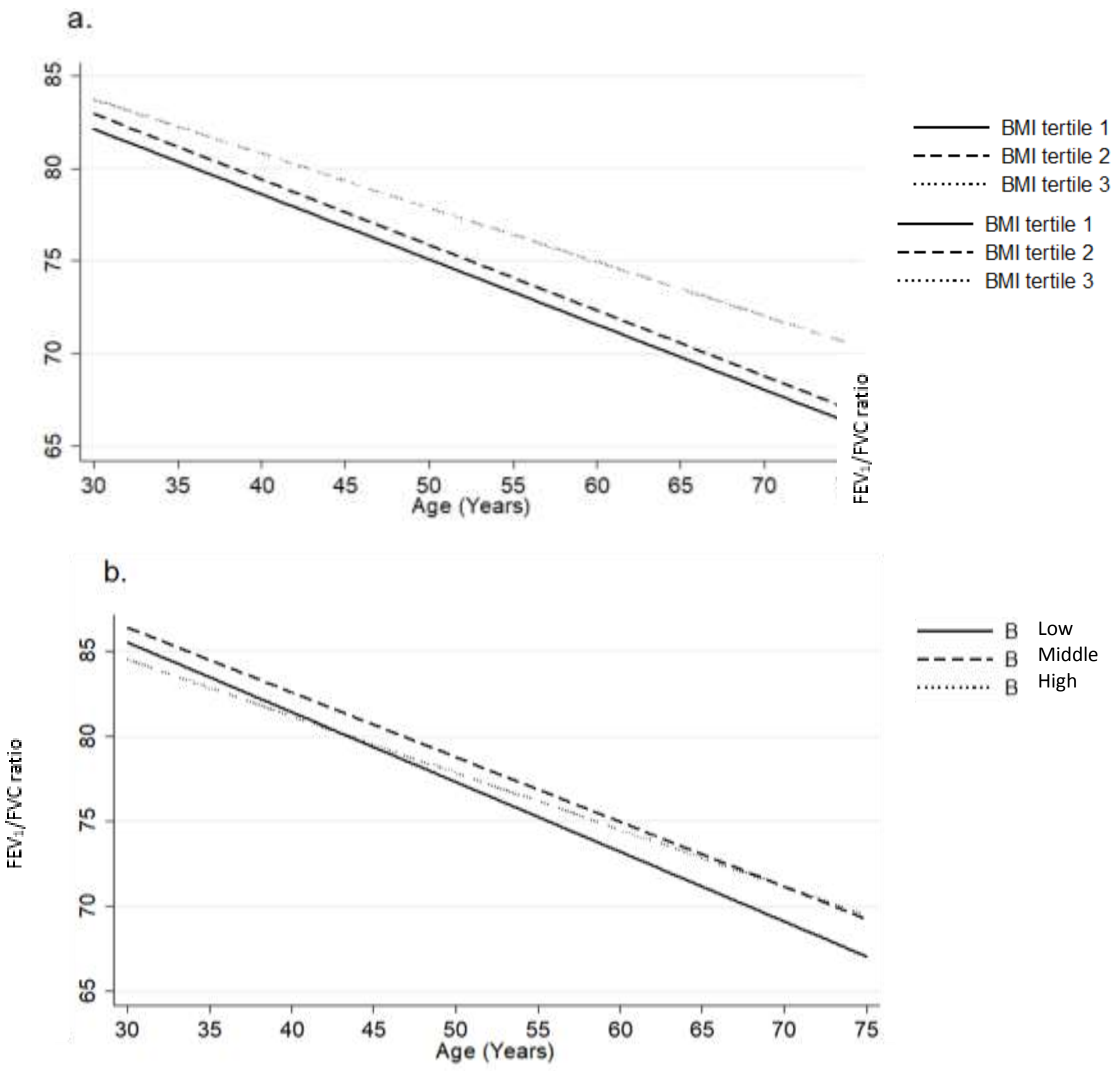

Figure S7: The change in FEV$_1$/FVC Ratio with age for BMI adjusted for pack years with participants who develop a restrictive pattern removed. a. men b. women

Abbreviations: FEV$_1$/FVC: ratio of the Forced Expiratory Volume in 1 second/Forced Vital Capacity, BMI: body mass index. Tertiles of BMI are based on individuals' mean BMI below age 40. Tertile 1 has the lowest BMI.

References

1. Kohansal R, Martinez-Camblor P, Agustí A, Buist AS, Mannino DM, Soriano JB. The natural history of chronic airflow obstruction revisited: an analysis of the Framingham offspring cohort. *Am J Respir Crit Care Med*. 2009;180(1):3-10.

2. Steiner MC, Barton RL, Singh SJ, Morgan MDL. Bedside methods versus dual energy X-ray absorptiometry for body composition measurement in COPD. *Eur Respir J*. 2002;19(4):626-631. doi:10.1183/09031936.02.00279602

3. Fairley L, Petherick ES, Howe LD, et al. Describing differences in weight and length growth trajectories between white and Pakistani infants in the UK: analysis of the Born in Bradford birth cohort study using multilevel linear spline models. *Arch Dis Child*. 2013;98(4):274-279. doi:10.1136/archdischild-2012-302778

4. Royston P, Altman DG. Regression Using Fractional Polynomials of Continuous Covariates: Parsimonious Parametric Modelling. *J R Stat Soc Ser C Appl Stat*. 1994;43(3):429-467. doi:10.2307/2986270

5. Tilling K, Macdonald-Wallis C, Lawlor DA, Hughes RA, Howe LD. Modelling Childhood Growth Using Fractional Polynomials and Linear Splines. *Ann Nutr Metab*. 2014;65(2-3):129-138. doi:10.1159/000362695

6. Tsao CW, Vasan RS. Cohort Profile: The Framingham Heart Study (FHS): overview of milestones in cardiovascular epidemiology. *Int J Epidemiol*. 2015;44(6):1800-1813. doi:10.1093/ije/dyv337
